# Perihematomal diffusion restriction as a common finding in large intracerebral hemorrhages in the hyperacute phase

**DOI:** 10.1371/journal.pone.0184518

**Published:** 2017-09-18

**Authors:** Tanja Schneider, David Frieling, Julian Schroeder, Jan Regelsberger, Gerhard Schoen, Jens Fiehler, Susanne Gellißen

**Affiliations:** 1 Department of Diagnostic and Interventional Neuroradiology, University Medical Center Hamburg-Eppendorf, Hamburg, Germany; 2 Department of Neurology, University Medical Center Hamburg-Eppendorf, Hamburg, Germany; 3 Department of Neurosurgery, University Medical Center Hamburg-Eppendorf, Hamburg, Germany; 4 Department of Medical Biometry and Epidemiology, University Medical Center Hamburg-Eppendorf, Hamburg, Germany; 5 Department of Diagnostic and Interventional Radiology, Schön Klinik Hamburg Eilbek, Hamburg, Germany; Henry Ford Health System, UNITED STATES

## Abstract

**Purpose:**

There is growing evidence that a perihematomal area of restricted diffusion (PDR) exists in intraparenchymal hemorrhages (IPH) within 1 week of symptom onset (SO). Here, we study characteristics and the clinical impact of the PDR in patients with hyperacute (≤ 6 hours from SO) IPH by means of apparent diffusion coefficient (ADC).

**Methods:**

This monocentric, retrospective study includes 83 patients with first-ever primary IPH from 09/2002-10/2015. 3D volumetric segmentation was performed for the IPH, PDR, and perihematomal edema (PHE) on fluid-attenuated inversion recovery, T2*/susceptibility weighted images, and ADC images.

**Results:**

A PDR was seen in 56/83 patients (67.5%) presenting with hyperacute IPH. Multivariate logistic regression analysis revealed every 10-year increase of age (HR 1.929, 95% CI 1.047–3.552, *P* = .035) and male gender (HR 5.672, 95% CI 1.038–30.992, *P* = .045) as significant predictors of the presence of a PDR, but not IPH size, IPH location, nor National Institutes of Health Stroke Scale Score (NIHSS) at admission. We found no difference in NIHSS at discharge, hematoma removal, or mortality rate in PDR-positive patients. ADC values of the PDR show a step-wise normalization with increasing time from SO.

**Conclusions:**

Occurrence of a PDR is a common finding in supratentorial hyperacute IPH, but shows no adverse short-term clinical impact. It may represent transient oligemic and metabolic changes.

## Introduction

Intracerebral hemorrhage or intraparenchymal hemorrhage (IPH) represents a second cause of stroke with high blood pressure being the main risk factor [1, 2]. On magnetic resonance imaging (MRI), 5 different stages of hematoma formation are recognized: hyperacute (≤ 6 hours from symptom onset, SO), acute (> 6 to 72 hours), early subacute (> 3 to 7 days), late subacute (> 1 to 2 weeks), and chronic (2 weeks to years) [3]. Due to lysis of red blood cells and changes in the oxygenation state of hemoglobin, all stages show a characteristic pattern on MRI [4, 5].

Typically, IPH can be surrounded by a vasogenic perihematomal edema (PHE) that appears hyperintense on fluid-attenuated inversion recovery (FLAIR) and apparent diffusion coefficient (ADC) maps. Within the PHE, the occurrence of patchy ADC hypointensities has been described [6–14]. 3 studies suggested that this phenomenon also occurs in hyperacute IPH, but no systematic study on this topic has been performed [12–14]. Furthermore, quantitative ADC measurements of the PHE area are lacking.

Here, we aim to determine the morphology and the clinical impact of the PHE region in hyperacute IPH patients by means of ADC. We hypothesize that ADC hypointensities are frequently found within the PHE and that this finding is associated with a worse clinical status and outcome.

## Materials and methods

### Study cohort

This single center, retrospective study was conducted in compliance with the local ethics committee (Ethik-Kommission der Ärztekammer Hamburg, WF-023/15) with a waiver of informed consent. We reviewed all patients ≥ 18 years in whom diagnosis of spontaneous first-ever IPH was made after an MRI was performed due to suspected acute ischemic stroke from 09/2002 to 10/2015 ≤ 6 hours from SO (hyperacute IPH). Patients with hemorrhagic conversion of acute ischemic stroke, traumatic IPH, underlying brain malignancy, and secondary causes of IPH (e.g. arteriovenous malformations and venous sinus thrombosis–since pathophysiology includes vessel occlusion and therefore a higher likelihood of ischemia) were excluded.

In total, 83 patients (54 males with a median age of 64 (interquartile range (IQR) 54.75–73.25) years and 29 females with a median age of 71 (IQR 58–80.5) years) met the inclusion criteria. IPH was classified as deep if the thalamus, basal ganglia, and/or internal capsule were suspected as bleeding origin. In a second step, electronic chart review of eligible cases was performed and medication, NIHSS on admission and discharge as assessed by the treating physician were recorded.

### Imaging protocol

MRI was performed at a 1.5 Tesla (Magnetom® Sonata, Siemens Healthcare, Erlangen, Germany; Magnetom® Symphony, Siemens Healthcare, Erlangen, Germany, and Magnetom® Avanto, Siemens Healthcare, Erlangen, Germany) in 82 cases or a 3 Tesla scanner (Magnetom® Skyra, Siemens Healthcare, Erlangen, Germany; Ingenia, Philips Medical Systems, Best, The Netherlands) in 1 case. Standard imaging protocol included an axial FLAIR and axial diffusion-weighted imaging (DWI, diffusion gradient b values of 0, 500, and 1000 s/mm^2^) with automated calculation of ADC maps in all cases. Furthermore, axial T2*-weighted gradient echo imaging (T2*) was performed in 71 cases and susceptibility weighted imaging (SWI) in 12 cases. Sequence parameters varied among the different scanners (Table 1). 42/83 patients underwent follow-up imaging within 10 days after SO (MRI = 1; computed tomography (CT) = 41 patients). Computed tomography (CT) was always performed on a 256-slice multi-detector CT (Brilliance iCT; Philips Medical Systems, Best, The Netherlands).

**Table 1:** Sequence parameters of FLAIR, DWI, and T2*/SWI of the different scanners.

MRI scanner	1.5 T			3 T
	Magnetom® Avanto	Magnetom® Sonata	Magnetom® Symphony	Ingenia
**No. of patients**	18	55	9	1
**DWI**				
TE (ms)	78	77	87	87
TR (ms)	2400	2600	3000	3549
Flip angle	90°	90°	90°	90°
Matrix	256x256	256x256	256x256	192x192
FOV	230x230	230x230	230x230	227x227
Pixel size	0.9x0.9	0.9x0.9	0.9x0.9	1.18x1.18
No. of slices	20	20	20	28
Slice thickness (mm)	5	5	5	4
Interslice gap (mm)	6.5	6.5	6.5	5
**FLAIR**				
TE (ms)	113	108	113	120
TR (ms)	7900	7900	7900	11000
TI (ms)	2356	2500	2500	2800
Flip angle	150°	150°	150°	90°
Matrix	384x512	384x512	384x512	400x400
FOV	172x230	172.5x230	172x230	230x230
Pixel size	0.45x0.45	0.45x0.45	0.45x0.45	0.57x0.57
No. of slices			20	28
Slice thickness (mm)	5	5	5	4
Interslice gap (mm)	6.5	6.5	6.5	5
T2*				
TE (ms)	40	59	72	14
TR (ms)	2460	2620	3160	651
Flip angle	90°	90°	90°	18°
Matrix	192x192	192x192	192x192	432x432
FOV	229x229	230x230	230x230	230x230
Pixel size	1.2x1.2	1.2x1.2	1.2x1.2	0.53x0.53
No. of slices	20	20	20	28
Slice thickness (mm)	5	5	5	4
Interslice gap (mm)	6.5	6.5	6.5	5
**SWI**				
TE (ms)	30			
TR (ms)	46			
Flip angle	15°			
Matrix	144x192			
FOV	144x192			
Pixel size	1.2x1.2			
No. of slices	64			
Slice thickness (mm)	2			

DWI, diffusion-weighted imaging; FLAIR, fluid-attenuated inversion recovery; FOV, field of view; SWI, susceptibility-weighted imaging; T, Tesla; TE, echo time; TI, inversion time; TR, repetition time

### Image analysis

Image Analysis was performed with Analyze Software System 11.0 (Biomedical Imaging Resource, Mayo Clinic, Rochester, MN, USA) by a single reader blinded to the patients status (D. F.) [15]. For this purpose, all sequences (FLAIR, ADC, and T2* or SWI) were spatially co-registered to one another using three-dimensional (3D) voxel registration module. If necessary, the registration was completed using manual 3D registration. Hence segmented regions of interest (ROIs) were transferable between all sequences.

**Fig 1:**
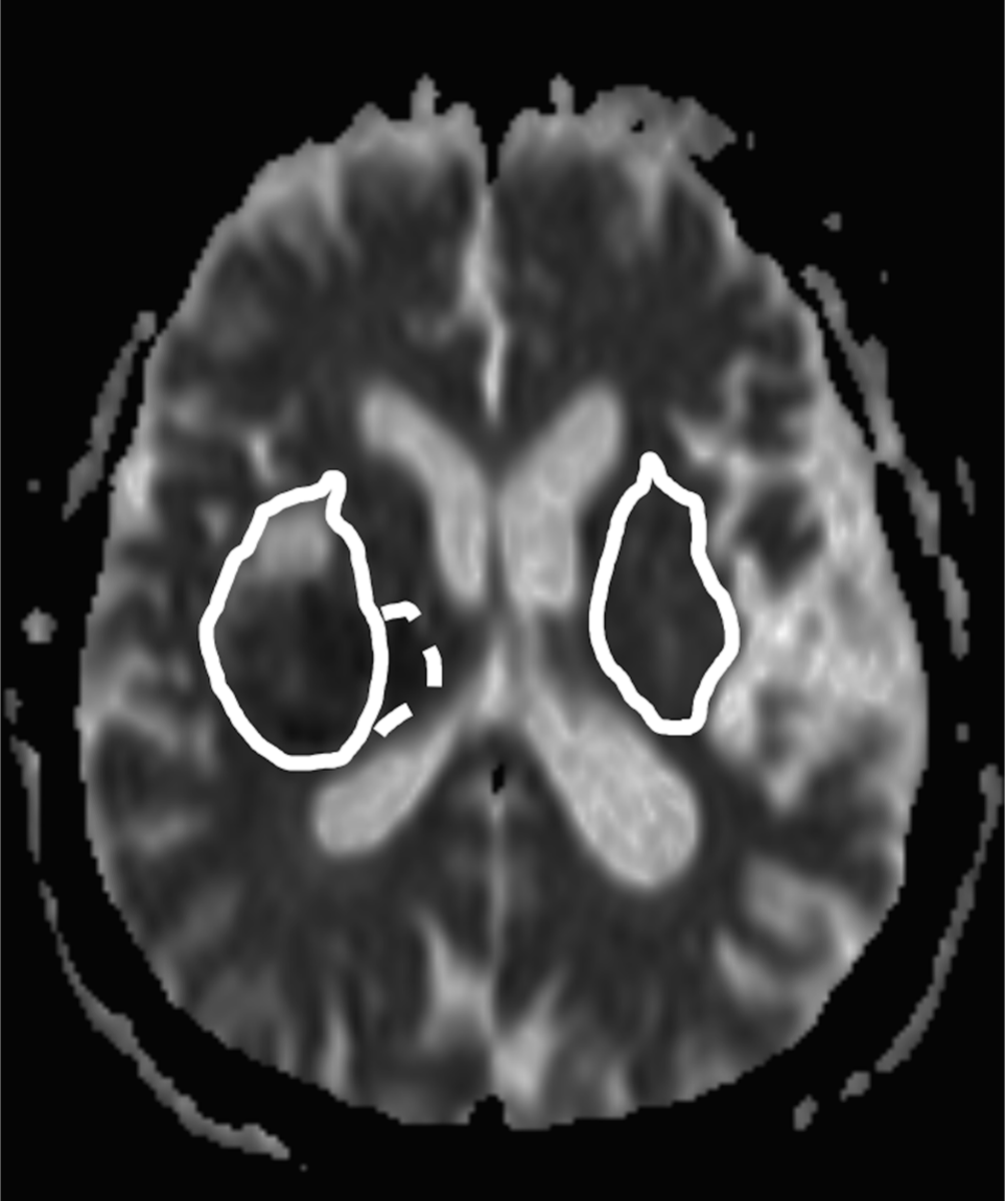
Example of the measurement technique of relative ADC values. The PHE ROI (solid line, right patient side) was transferred to a mirror region (left patient side) and then manually adjusted. Median absolute ADC values of the PHE (solid line), PDR (dotted line), and IPH (not shown) were each divided by median ADC values of the mirror region to calculate the corresponding relative ADC value.

First, the parenchymal hematoma was contoured manually on all T2* or SWI images. Since T2* and SWI images have been shown to overestimate the size of the hematoma by approximately 20%, the co-registered FLAIR was used to precisely define the borders of the clot (IPH ROI) [16]. Afterwards, the whole lesion, defined by the borders of the PHE, was segmented on FLAIR images (lesion ROI), Fig 1. IPH volume directly corresponded to the IPH ROI (in cc), whereas for calculation of PHE volume IPH ROI was subtracted from lesion ROI (lesion ROI–IPH ROI = PHE ROI, in cc).

**Fig 2:**
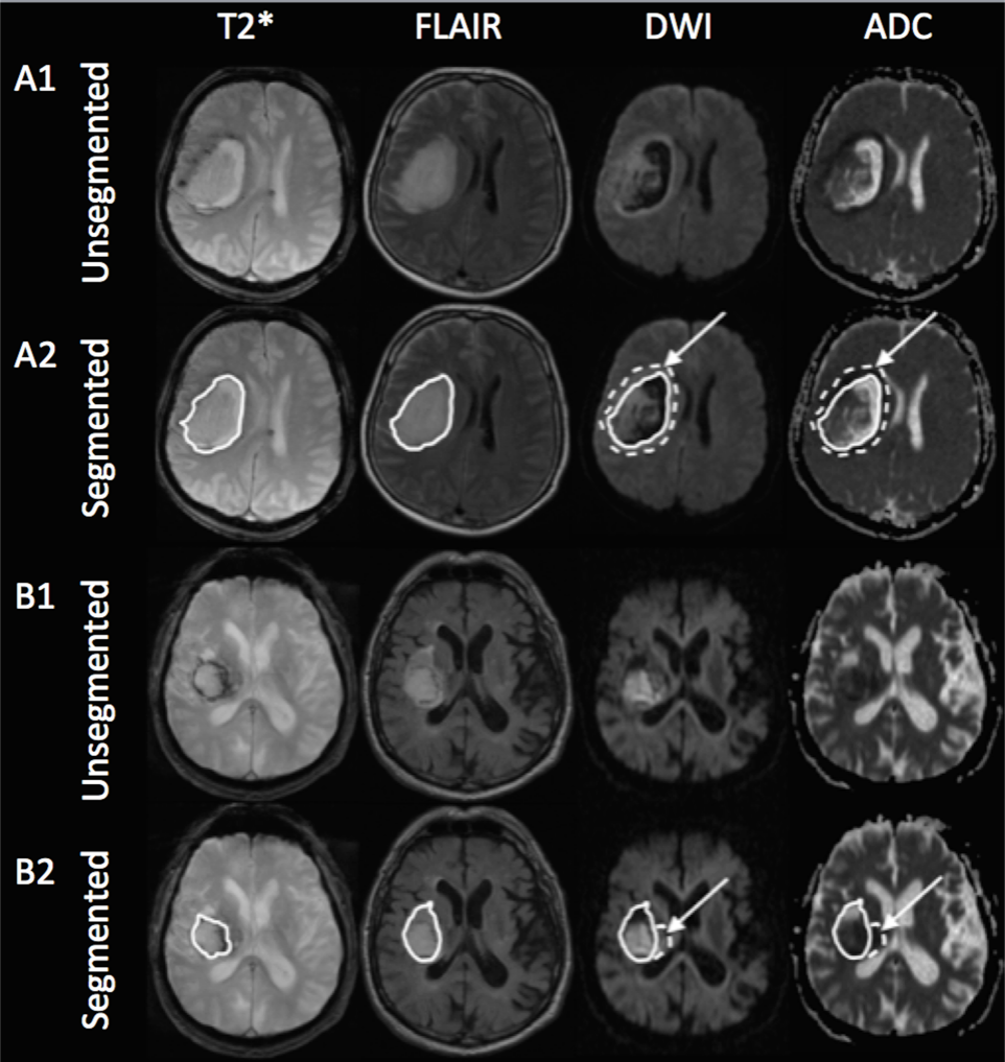
Two examples of unsegmented (A1 and B1) and segmented (A2 and B2) hyperacute hypertensive right-sided intraparenchymal hemorrhages (IPH) on co-registered axial T2*, fluid-attenuated inversion recovery (FLAIR), diffusion-weighted imaging (DWI), and apparent diffusion coefficient (ADC) images. Example A shows the MRI of a 51 year old male patient 1.5 hours after symptom onset and example B that of a 82 year old male patient in a 3-hour window. The solid line on T2* (A2 and B2) shows the unadjusted IPH ROI and that on FLAIR, DWI, and ADC the adjusted whole lesion ROI (IPH+PHE, IPH ROI is not shown here for the sake of clarity). The dotted line with arrows on DWI and ADC represent the PDR ROI.

In a majority of cases, we found a rim of restricted diffusion adjacent to the outer border of the PHE. This perihematomal rim (PDR) was defined as hyperintense area on b1000 images and hypointense on the corresponding ADC map applying a threshold of ≤ 1.5 standard deviations of the median ADC value in a manually corrected mirror region (Fig 1). In cases a PDR was present, it was segmented on ADC images. For calculation of relative ADC values (rADC), median absolute ADC values of IPH, PHE, and PDR were each divided by median ADC values of the manually drawn corresponding contralateral mirror region. Two segmentation examples are shown in Fig 2.

Accuracy of lesion masks was evaluated by 2 independent readers (S. S. and T. S. with 10 and 4 years of experience in Neuroradiology, respectively). For IPH, edema, and PDR ROI, P25 (first quartile), mean, median, and P75 (third quartile) ADC values were read and volumes were computed. Follow-up images were visually assessed by S. S. and T. S. in consensus and patients were classified into 2 groups, based on whether the IPH decreased or increased in size during the first 10 days after SO.

### Statistical analysis

Pearson’s correlation was used to compare IPH and PDR volumes. The differences in age, IPH volume, NIHSS at admission and at discharge among PDR-positive and PDR-negative patients, differences in PDR volumes by location, PDR/IPH volumes, and absolute ADC values in patients with and without IPH growth within 10 days after SO, in patients with and without hematoma removal, and patients who died were compared by Mann-Whitney U test. Pearson’s chi-squared test was performed to test gender frequency, time from symptom onset to MRI, antiplatelet therapy status, and the frequency of lobar and deep IPH in PDR-positive and PDR-negative patients. Fisher’s Exact test was used to determine differences of the frequency of a PDR in supra- and infratentorial locations, hematoma removal rate, mortality rate, anticoagulation therapy status, and the frequency of IPH volume increase within 10 days after SO. Differences in PDR size and time from symptom onset to MRI were determined by using the Kruskal-Wallis test.

For multivariate analysis, a logistic regression was performed including gender, age, IPH location, time from SO to MRI, NIHSS at admission, and log IPH volume. A *P* value < .050 was considered significant. Data collection was performed using IBM SPSS Statistics®, version 20 (IBM® 2011, Armonk, NY, USA). If not otherwise indicated, data are given as median and IQR (in parenthesis).

## Results

### Descriptive statistics

Baseline patient characteristics are given in Table 2. IPH location was supratentorial in 81 cases (66 deep, 15 lobar) and infratentorial in 2 cases (1 cerebellar, 1 pontine). MRI was performed after 2.0 (1.0–2.75) hours from SO. In 15 patients, it was acquired within 6 hours from SO (assured last seen well-time below 6 hours), but exact SO was not observed („wake-up IPH“, only supratentorial IPH).

**Table 2:** Baseline characteristics of all patients and perihematomal rim (PDR)-positive/PDR-negative patients. Data are given as median and interquartile range (in parenthesis).

	All patients (n = 83)	PDR+ patients (n = 56)	PDR- patients (n = 27)	*P* value
Age (in years)	65 (56–78)	64 (56–73.8)	70 (60–80)	0.175
Gender				
• Male	54	34	20	0.232
• Female	29	22	7	
IPH location				
• Supratentorial	81	56	25	0.103
• Infratentorial	2	0	2	
IPH volume (in cc)	12.9 (7.9–26.6)	16.6 (8.2–37.3)	9.9 (6.9–15.5)	0.021
Time from SO (in hours)				
• ≤1	19	14	5	
• >1-≤2	32	21	11	
• >2-≤3	10	9	1	
• >3-≤4	3	3	0	
• >4-≤5	2	2	0	
• >5-≤6	2	2	0	
• “Wake-up”	15	5	10	
NIHSS at admission	11 (7–15)	12 (8–15)	9 (7–12)	0.072
NIHSS at discharge	7 (3.5–11)	7 (3–11)	7 (4–9)	0.872
Hematoma removal rate	4 (4.8%)	4 (7.1%)	0	0.299
Mortality rate	7 (8.4%)	5 (8.9%)	2 (7.4%)	0.711

IPH, intraparenchymal hemorrhage; NIHSS, National Institutes of Health Stroke Scale; PDR, perihematomal rim of restricted diffusion; SO, symptom onset

### Perihematomal rim (univariate analysis)

56/83 patients (67.5%) patients showed a PDR of low ADC, as exemplified in Fig 2, measuring 1.98 (0.97–4.74) cc with no predilection for gender (χ(1) = 1.430, *P* = .232), age (U = 616.5, *P* = .175), or higher systolic blood pressure (U = 729, *P* = .894). PDR occurred at any time from SO (Table 2) and never appeared completely hyperintense on FLAIR by visual inspection (Fig 1). A PDR was observed in supratentorial IPH only (*P* = .103), with an equal distribution among lobar (n = 15/81) and subcortical grey matter (basal ganglia) bleedings (n = 66/81, χ(1) = 2.651, *P* = .103). Lobar PDR (3.24 cc, 1.90–6.40 cc) were greater in size compared to subcortical grey matter PDR (1.31 cc, 0.95–4.34 cc), but the difference was not significant (U = 182, *P* = .058). We found a significant, positive correlation between PDR and IPH volume (r = 0.652, *P* < .001). PDR volume was equal over time (χ2(5) = 7.544, *P* = 0.183). There was no difference in anticoagulation or antiplatelet therapy status between PDR-positive and PDR-negative cases (*P* = .418 and *P* = .434, respectively).

### Distribution of ADC values of the PDR

**Fig 3:**
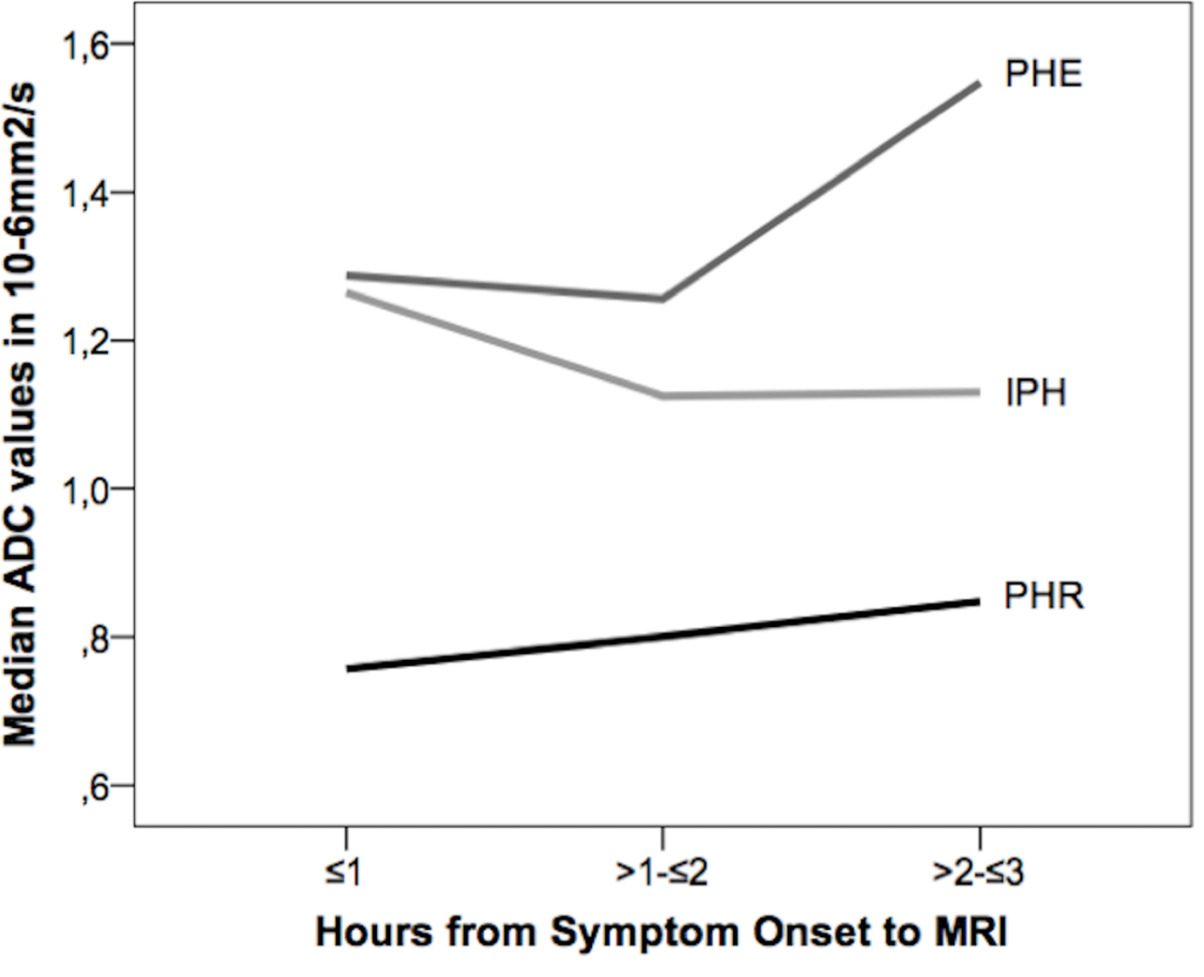
Comparison of relative median ADC values of IPH, PDR, and PHE 0-≤3 hours after symptom onset. Over time, relative PDR and PHE ADC values rose whereas relative IPH values declined.

Absolute ADC values within the PDR were 624.5*10^-6^mm^2^/s (598.5–670*10^−6^ mm^2^/s) and therefore lower than those measured in IPH (789*10^-6^mm^2^/s, 710–890*10^-6^mm^2^/s) and PHE (1030*10^-6^mm^2^/s, 948–1110*10^-6^mm^2^/s). Association of absolute PDR-ADC values with time from SO showed a step-wise increase in P25, mean, median, and P75 up to 3 hours. „Wake-up”IPH showed PDR ADC values similar to patients imaged between >3-≤4 hours, but interquartile ranges (P25-P75) of „wake-up”IPH cases were 2 to 3 times greater than those with known SO. When excluding „wake-up”IPH cases and cases imaged > 3 hours from SO due to low patient numbers, P25, mean, median, and P75 absolute PDR-ADC values showed a step-wise increase, but the difference was not significant. rADC values for PHE and PDR rose from 0-≤3 hours after SO whereas rADC values for IPH declined. PDR-rADC values always remained below 90%, whereas PHE-rADC values always always stayed above 110% (Fig 3).

### Outcome measurements

Median hospital stay was 16 (9–21) days. Median NIHSS for supra- and infratentorial IPH at initial presentation was 11 (8–15) and 4.5 points and at discharge from hospital 7 (4–11) and 3 points, respectively, without a difference in NIHSS between patients with and without PDR (initial NIHSS: U = 560.5, *P* = .072; NIHSS at discharge: U = 561.5, *P* = .872). Cases with higher IPH volumes showed significantly higher NIHSS at admission (r = 0.302, *P* = .006) and at discharge (r = 0.293, *P* = .012). Of the 41 patients who underwent follow-up imaging within 10 days after SO, 4 showed hematoma enlargement that was neither associated with presence of a PDR (*P* = .129). We found no difference in absolute IPH, PHE, and PDR ADC values in patients with and without IPH growth (U > 7.5, *P* > .571). 4 patients underwent hematoma evacuation due to life threatening mass effect with consecutive impairment of cerebrospinal fluid flow immediately following admission. Those patients neither showed a significantly higher initial PDR (U = 85, *P* = .570) nor IPH volume (U = 77, *P* = .087) compared to conservatively treated cases, and we found no difference in ADC distribution of the PDR between both groups (*U* > 95.5, *P* > .794). 7/83 patients died after 18 (3tabl-27.5) days from SO regardless of the presence of a PDR (*P* = .711). Deceased PDR-positive patients had higher PDR and IPH volumes than PDR-negative cases (U = 104.5, *P* = .098 and U = 68, *P* < .001, respectively), but there were also no differences in absolute ADC values (U > 103.5, *P* > .093).

### Perihematomal rim (multivariate analysis)

In multivariate logistic regression analysis adjusted for gender, age, IPH location, time from SO to MRI, NIHSS at admission, and log IPH volume, male gender (hazard ratio (HR) 5.672, 95% confidence interval (CI) 1.038–30.992, *P* = .045) and each 10-year increase of age (HR 1.929, 95% CI 1.047–3.552, *P* = .035) were identified as independent risk factors for the occurrence of a PDR (Table 3).

**Table 3:** Multivariate regression analysis with gender, age, IPH location, time from symptom onset to MRI, NIHSS at admission, and log IPH volume as coefficients.

Coefficient	HR	95% CI	*P* value
Gender (male vs female)	5.672	1.038–30.992	0.045
Age (10-year interval)	1.929	1.047–3.552	0.035
Location (deep vs lobar)	4.294	0.329–55.960	0.266
Time from SO to MRI (1-hour interval)	0.388	0.144–1.048	0.062
NIHSS at admission	0.969	0.827–1.135	0.695
Log IPH volume	0.910	0.136–6.084	0.922

CI, confidence interval; HR, hazard ratio; IPH, intraparenchymal hemorrhage; NIHSS, National Institutes of Health Stroke Scale Score; MRI, magnetic resonance imaging; SO, symptom onset

## Discussion

ADC is a measure of the diffusibility of water molecules within a tissue and is determined from DWI. Low ADC values correspond to restricted water diffusion and are considered to be caused by cell swelling or increased cellular density [17]. In hyperacute IPH patients, evidence for diffusion restriction in the periclot area was shown in 3 studies: *Kidwell et al*. first described a rim of decreased ADC values in a subset of 3/12 patients, but did not acquire FLAIR sequences. Therefore, it becomes not entirely clear whether the rim is part of the edema or is entirely located outside the edema [13]. *Schellinger et al*. reported 7/32 patients with lower ADC values/cytotoxic edema within a ROI of 1 cm surrounding the IPH compared to the healthy hemisphere [14]. The most recent study by *Stösser et al*. showed a PDR in 88/172 (51.2%) IPH patients within 24 hours of symptom onset, but they did not separately examine the subgroup of patients in a hyperacute or 6-hour time window [12].

The MRI pattern of low ADC without corresponding hyperintensity on FLAIR is well-known from ischemic stroke patients in the first few hours with the tissue being hypoperfused, but potentially salvageable from ischemia. But do the same mechanisms causing brain damage in ischemic stroke occur in the PDR? Since perfusion deficits were shown to occur in the ipsilateral hemisphere rather than within the perihematomal area *(Kidwell et al*.*)* while no correlation between cytotoxic edema and prolongation of mean transit time was found *(Schellinger et al*.*)*, both authors conclude that the rim/ROI of decreased ADC primarily represents temporary depression of regional neuronal metabolism rather than true ischemia. Comparable conclusions were drawn from perfusion/diffusion studies of acute and hyperacute ischemia [6–9]. Additionally, a positron emission tomography study demonstrated that oxygen extraction fraction (OEF) is reduced in acute IPH as opposed to ischemia, where OEF is increased [18]. Our results and the work of *Stösser et al*. further support this hypothesis: we were not able to show a difference in NIHSS at admission and discharge from hospital in patients with and without PDR, which would be expected if the PDR represents a frank ischemic region. Additionally, the finding that cases with higher PDR volumes showed significantly higher NIHSS scores at admission and discharge and had an increased mortality seems to be more attributable to higher initial IPH volumes. However, in contrast to our results, *Orakcioglu et al*. did not find perihemorrhagic ADC decreases in hyperacute IPH in a rat model [19].

Quantitative ADC values of the PDR were not studied before. We showed a continuous increase of absolute ADC values and rADC for up to 3 hours after SO (when excluding „wake-up”IPH cases and cases imaged >3 hours from SO due to low patient numbers). Therefore, diffusion restriction within the PDR is most pronounced directly after IPH onset and seems to diminish with time. This might be an explanation why other earlier reports studying the acute and subacute IPH phase (SO > 6 hours) did not describe a PDR. The PDR might primarily be related to a number of inflammatory actions and excitotoxic reactions caused by the blood clot [20]. Whether the overall decrease of PDR-ADC values within >3-≤6 hours from SO is due to the low patient number or not should be evaluated in further studies.

There are some limitations to our study. First, we may have missed cases that were suspected of hemorrhagic stroke (e. g. patients who did not show an abrupt SO or patients whose symptoms had not reached maximum intensity at SO) by MRI, because in our institution, these patients receive a computed tomography as initial imaging modality at admission. However, since MRI was routinely performed in patients with suspected stroke in a 6-hour time window, we do not assume a greater selection bias here. Secondly, due to the retrospective character of the study, sequence parameters differed by scanner and ADC values may not be directly comparable. Even though there is no common DWI quality assurance protocol, a study of Belli et al. showed a good agreement of a phantoms mean ADC value in a multicenter comparison [21]. In this study, we used relative ADC values/ratios that helped eliminating differences in the equipment. We did not assume systematic errors in the segmentation of partly small PDR volumes since we had 3 readers and MRI was shown to be very accurate in determining even minor tumor volumes [22]. Furthermore, perfusion imaging was not routinely performed after diagnosis of IPH in MRI; we were therefore not able to study perfusion changes in the PDR. Additionally, our patients did not routinely undergo a follow-up MRI (since CT is sufficient) that would have helped to further determine the FLAIR characteristics of the PDR in the acute or subacute period. Third, the number of patients imaged between 3 and 6 hours was too low to draw final conclusions about the PDR composition in this phase. Fourth, depending on the underlying sequence, an IPH typically shows different volumes (for example, SWI tends to overestimate IPH sizes, called “blooming effect”) [16]. However, we accounted for this problem by spatial registration of SWI and ADC images after the predefinition of IPH ROI on SWI and subsequent careful manual correction of the ROIs, if applicable.

In conclusion, this is the first study precisely examining the perihematomal region in patients with hyperacute IPH by means of DWI/ADC mapping. We found a PDR adjacent to the perihematomal edema without corresponding FLAIR hyperintensities in a majority of patients. The degree of diffusion restriction was most pronounced directly after IPH onset and significantly declined in the following hours. Whether this phenomenon is caused by reversible hypoperfusion and/or metabolic changes has to be further studied to better understand pathophysiological processes involved in early IPH formation. Nevertheless, occurrence of PDR is not associated with worse clinical status and is therefore not a predictive marker for clinical status or short-term outcome.

## Supporting information

S1 Table 83 patients hyperacute ICH. (SAV)
